# Leadership has been happening

**DOI:** 10.1590/1806-9282.20241245

**Published:** 2024-11-11

**Affiliations:** 1 Hospital Santa Maria, Department of Internal Medicine – Lisbon, Portugal.

From a humanist perspective, leadership is the exercise of power, understood as an act of sharing, leading to the attainment of the "common good." Power as "potentiality," whose only limitation is the existence of other people, since it will correspond to the human condition of plurality, as stated by Hanna Arendt, insofar as it "springs up between men when they act together and vanishes the moment they disperse"^1^. This power is equivalent to leadership as a process, since "[t]he only indispensable material factor in the generation of power is the living together of people"^1^, which is seminal for the success of a full leadership path.

The search for a definition of "Leadership" risks being reductive and partial, and in the light of countless attempts over the decades^2^, a (near) impossibility. The parallelism with "power"— as outlined by Hanna Arendt^1^— can be a synthesis that fits the vision of leadership focused on the shared livingness of responsibilities for the attainment of individual and collective well-being.

Looking at the seventeen Demitras leadership styles^3^, the difficulty of defining leadership and restricting its scope to a single model is evident, since the non-conflicting intersections between leadership styles are countless but desirable. Not having proactively adopted specific leadership tools or models, embodied in an explicit theoretical framework, now in the attempt to elaborate a structured point of view, I find that the principles of creative transformation and authenticity have echo in the well-studied models "Transformational" and "Authentic Leadership."

The four components of Transformational Leadership^4^ translate a structure of societal values that reflect the plural, humble, and humanistic vision:

1. Idealized Influence is anchored in trust and respect;
2. Inspirational Motivation is vivid with enthusiasm and optimism;
3. Intellectual Stimulation is that which promotes creativity and innovation, and
4. Individualized Consideration is paradigmatically focused on giving attention to others and on transparent communication.

The combination of these pillars with the principles of genuineness, sincerity, truth to oneself, and ethics that characterize "Authentic Leadership"^5^ creates the conditions for the success of organizations to be morally sustainable. Authenticity is assumed as a basic element for a set of leadership theories that are centered on a humanistic dimension and on respect for others in human relationships, where the leader emerges as a "Moral Agent," facilitating just, noble, and legitimate actions taken by the elements of the organization^6^.

Threats to plural democracy are multiplying every day, and relational oversimplification is exploited as a true matrix for the future of societies. To counteract the real and growing complexity inherent to modern democracies^7^, the factors that make up the construct of Authentic Leadership (Self-Awareness; Relational Transparency; Balanced Processing; Internalized Moral Perspective)^6^ are particularly relevant as engines for sustaining a righteous humanist pluralism. Leaders have the duty, even a moral one, to contribute to the survival and affirmation of the principles of democracy, without compromising the pursuit of the success of organizations. From this combination of values, commitments, and actions, it will be possible to move from "attempted leadership"—with an individual focus—to "successful leadership"—where interpersonal relationships are already established—and then culminate in "effective leadership"—which entails both a mutual and a shared gain^8^.

The principles and values underlying Transformational and Authentic Leadership find their paradigmatic space of application in health organizations, since here, the design of the common good, the focus on others, and humanistic altruism are the central elements of their actions. The profile of highly differentiated competencies that characterizes health agents, with autonomy of action and of decision-making, additionally supports the alignment with leadership strategies focused on open communication, stimulation of innovation and creativity, respect, and promotion of initiative and professional autonomy, as well as transparency and assertive humility.

In paths embracing and practicing leadership, thinking about the whole and looking at the organizational comprehensiveness must always be concerns and stimuli underlying the leadership experiences with the unavoidable purpose of the satisfaction of contributing. To contribute to the attempt of bringing improvements to the organization and the community, to contribute to the affirmation of authenticity, trust, and transparency as foundational elements of the organization's journey.

The experience in complex organizations, particularly in times of crisis, stimulates reflection about each of the organizational agents, with particular emphasis on those who assume transversal responsibilities. Enduring the stormy pandemic turbulence in medical leadership roles was a monumental challenge, with responsibilities that came with increasing demands to and from everyone, sometimes even more than would be thought possible, and with an acute sense of the need for balance and justice, but also for clarity, conviction, and trust. The search for virtues such as vigor, passion, temperance, or citizenship^9^, supported by the conviction of the essentiality of looking at oneself in an incessant critical analysis of self-awareness measured by the vision of others^10^, have been and will continue to be determining factors of a leadership that transitions from success to effectiveness.

That was a time when the importance of constructive doubt, creative dialectics, but also of assertive decision-making, asserted themselves as paradigms^11^. At the same time, the affirmation of "no" as a path to "yes," what William Ury designates as a "positive no,"^12^ proved to be a precious instrument of leadership, guiding the escape from the triple trap of accommodation, attack, and abstention.

Surprisingly, the post-pandemic aftermath does not seem to have brought the reinforcement of participatory, creative, and shared aspects in the leadership processes of health organizations. We are witnessing the (re)affirmation of the transactional and mechanistic aspects, where autonomy is presented as a banner but is not objectified in organizational processes. What could be the "transformational revolution" in the health system has been held hostage by a communicational fallacy that leads us to question how real this inauthentic reality is^13^.

Looking at the present environment and context of health organizations, I am dominated by the feeling of disappointment. Disappointment with the realization that, not only at the societal level but in the highly differentiated, demanding, and intellectually dense environment of health organizations, predominate the most basic, primordial, and simplistic instincts. The emphasis on immediacy and self-centralism dominates not only the decision-making processes but also interpersonal and intersectoral relationships. Honesty and transparency in relationships, the valorization of knowledge in decision-making, and the primacy of moral values over individual (or sectorial) interests are components that are submerged by the suffocating perpetuation of the protective boundaries of micro-powers and by the inability to look at the other, under the penalty of becoming universally evident the weaknesses of those who can only prevail in the obscurantism.

It is with discouragement and hopelessness that I see the rampant structural transactionality in health organizations and it being reinforced, where each one, more and more, seeks their own return, and in this fall the system sinks.

Nonetheless, I maintain the hope and the determination that authenticity can prevail and succeed in overcoming the crisis of confidence that undermines [the] health [of our democracies]. Leaders should have the ability to promote the trust that derives from the supreme act of delegation while empowering others as a corollary of the development of skills, ensuring follow-up for the joint achievement of goals, and promoting, in an adaptive way, the alignment of attitudes^14^.

To paraphrase Ricardo Vargas, "Leadership doesn't exist, it happens."^14^
